# Focus on the notice: evidence of spatial skills’ effect on middle school learning from a computer simulation

**DOI:** 10.1186/s41235-020-00263-0

**Published:** 2020-11-25

**Authors:** Colleen M. Epler-Ruths, Scott McDonald, Amy Pallant, Hee-Sun Lee

**Affiliations:** 1 Shikellamy High School, Sunbury, PA 17801 USA; 2 Pennsylvania State University, University Park, USA; 3 Concord Consortium, Concord, USA

**Keywords:** Spatial skills, Plate tectonics learning, Computer visualizations, Noticing

## Abstract

This article represents the findings from the qualitative portion of a mixed methods study that investigated the impact of middle school students’ spatial skills on their plate tectonics learning while using a computer visualization. Higher spatial skills have been linked to higher STEM achievement, while use of computer visualizations has mixed results for helping various students with different spatial levels. This study endeavors to better understand the difference between what high and low spatial-skilled middle school students notice and interpret while using a plate tectonic computer visualization. Also, we examine the differences in the quantity and quality of students’ spatial language. The collected data include student spatial scores, student interviews, screencasts, and online artifacts. The artifacts were students’ answers to questions inserted in an online curriculum (GEODE) with the embedded computer visualization (Seismic Explorer). Students were asked what they “noticed” during interviews and in the curriculum. Typed student answers and interviews were analyzed for types and quantity of spatial words. Analysis of typed answers and interviews indicated that there are differences in the number and types of spatial words used by high or low spatial students. Additionally, high spatial learners talk about depth, notice patterns in data and are more likely to make a hypothesis to explain what they see on the screen. Findings suggest that students go through an iterative cycle of noticing and interpreting when using a scientific model. Overall, results show a significant positive relationship between spatial skills and what students notice while learning plate tectonics. An explanation for the increased gain in plate tectonics comprehension is that students with higher spatial skills notice more, so they are able to interpret more details of the model. This finding implies that students with low spatial skills do not benefit as much from use of a computer visualization and will need more scaffolding in order to interpret details in the computer visualization.

## Background

Thinking spatially is complex as it involves various aspects of spatial abilities including mental rotation of objects, perception of one’s location in a setting, and visualization of how material would fold or unfold (Liben and Titus 2012; National Research Council 2006). There is no consensus on how to classify “spatial thinking.” While spatial thinking covers a variety of cognitive processes (Kozhevnikov and Hegarty 2001; McGee 1979; Wang 2017), “spatial skill” relates to a specific category within spatial thinking and represents the cognitive processes of mental rotation, defined as the ability to quickly and precisely rotate either 2D or 3D figures; spatial orientation, defined as the ability to find a spatial relationship in reference to as student’s body, and spatial visualization, defined as ability to perform a complex spatial procedure with many steps, perspective taking and so on (Liben, personal communication, October 26, 2018). Rather than ability or intelligence, the term skill is used to emphasize that it can be improved through training, learning or experience (Newcombe 2010; Uttal et al. 2013a, b).

Research on spatial skills indicates an important link between being able to think spatially and understanding complex scientific content, in particular geosciences (Kastens and Ishikawa 2006; Kastens et al. 2009; Manduca and Kastens 2012; Uttal and Cohen 2012). Geoscience is considered a spatial science, making it difficult for novices who cannot discern spatial properties to learn earth science content (Liben and Titus 2012). Studies show that students who are not adept at spatial visualization, perspective taking, and mental rotation are less successful in science, technology, engineering, and math (STEM) classes and tend to leave those majors (Uttal and Cohen 2012). Most studies of spatial skills concerning science learning were conducted in the undergraduate classrooms (Uttal et al. 2013a, b). Less research has been conducted at the secondary school level with regard to spatial skills and STEM learning (Newcombe 2010, 2013). Research in K-12 science learning found that prior knowledge and reasoning (Keig and Rubba 1993) and spatial ability (Klein and Koroghlanian 2004; Ganley et al. 2014) help students learn science content. In order to improve instruction and enhance students’ achievement, researchers need to have a better understanding of how spatial skills are related to science learning across students K-12.

Computer visualizations are often used in K-12 science classrooms to supplement curriculum and may require students to use a variety of spatial skills. Usually embedded in an inquiry science learning environment (Linn et al. 2010), dynamic visualizations, which are defined as digitally animated, interactive representations, expose certain aspects of the scientific phenomenon for the learner’s manipulation (Renken et al. 2016) and allow the learner to understand inner workings of a complex system such as a Earth’s climate (Pallant and Lee 2014). Fortunately, studies indicate that computer visualizations can help students mitigate issues with spatial understanding (Höffler 2010;Rutten et al. 2012; Smetana and Bell 2012) by making abstract concepts more concrete, simplifying parts of the world that are too complex to visualize, and lowering the cognitive load of visualizing other complex phenomena. Cognitive load refers to the degree to which working memory can be overloaded with information to be processed and remembered by the brain (Baddelely and Hitch 1974; Sweller 1988). Cognitive load can be reduced by 3D and dynamic visualizations to compensate for students low spatial skill (Höffler 2010; Lee 2007). However, other studies hypothesize that learners with high spatial abilities generally develop deeper understanding from dynamic visualizations perhaps due to “ability-as-enhancer,” their high spatial skill enhances good instruction or “ability-as-compensator,” where high spatial skill compensates for poor instruction, because high spatial students need fewer cognitive resources to hold mental pictures (Mayer and Sims 1994; Hays 1996; Hegarty and Sims 1994). Since computers have become standard classroom resources in the USA, computer-based dynamic visualizations have become more available. Research is needed to explicate why different learning outcomes emerge for students who are spatially adept and those who are not while using dynamic 3D computer visualizations in the science classroom (Hoffler 2010).

Researchers have tried to understand how spatial skills can be evaluated and framed in the context of student learning when supported with computers. Science education researchers (Carey 1986; Chi et al.^1981^; Jacobson 2001; Petcovic and Libarkin 2007) have used aspects of novice/expert differences to study spatial skills in science. Professional vision (Goodwin 1994) describes how experts notice important subtleties that are elusive or invisible to novices. One way to gauge developing professional vision with spatial skills is through examining the *noticing* effect (Lindgren and Schwartz 2009) by asking what students notice on a computer screen. The noticing effect is especially useful for determining levels of student understanding. Since learning is often defined as developing knowledge that allows for formulating inferences beyond the given information (Bruner 1957; Goodwin 1994), spatial skills will likely be associated with what students notice and, in turn, make inferences.

This article describes the qualitative results from a project that included a pilot study and main study addressing these issues. In the pilot study, we first determined the spatial affordances in the computer visualization used in the research, Seismic Explorer. Seismic Explorer is embedded in the GEODE curriculum, which is designed to support student learning around the patterns between volcanoes, earthquakes and plate tectonics. We then collected student answers to noticing questions placed within the online GEODE curriculum. The initial student answers were used to developed a Spatial Noticing Coding Framework to code the spatial characteristics of students’ typed answers. In the second (main) study, we investigated the intersection of student spatial skills based on the Spatial Reasoning Instrument (Ramful et al. 2017) with what students noticed in interviews and in typed answers in curriculum while using the Seismic Explorer computer visualization during a middle school earth science class. The qualitative data were collected and analyzed to answer these research questions:

- What are the differences in what high or low spatial students notice and interpret while using the computer visualization?
- Does the quality and quantity of spatial language use differ between high and low spatial students?

## The learning context

The computer-based visualization used in this study was part of an online middle school curriculum module focused on the guiding question, “What will Earth look like in 500 million years?" The module was developed as part of Geological models for Explorations of Dynamic Earth (GEODE) project. The GEODE module was designed to help students understand plate tectonics from the system perspective, an idea that tectonic plates cover the entire surface of the Earth and are in constant motion, causing interactions in specific ways along all of their boundaries. Students investigate several cases of plate boundary types and formulate hypotheses about causal mechanisms leading to seismic activities and landforms found on Earth. The plate tectonics module takes five to eight class periods to finish and contains two computer-based simulation/visualization tools: the Seismic Explorer for interactively retrieving volcanic and earthquake data around the world over the last eighty years and Tectonic Explorer for simulating plate motions on an “Earth-like” planet. This study focuses on data visualizations shown on the Seismic Explorer.

Seismic Explorer (SE), developed by Concord Consortium, is a web-based data visualization tool used to model earthquake, volcano, and plate data onto an Earth map (Pallant et al. in press). SE is a dynamic visualization (Linn et al. 2010) embedded in the plate tectonic curriculum. SE is a robust application that provides visualization requiring many spatial skills: spatial orientation, mental rotation, and spatial visualization. Students must use these skills to be able to interpret the spatial nature of SE to have the most sophisticated understanding possible of the earthquake/volcano/plate data.

**Fig. 1:**
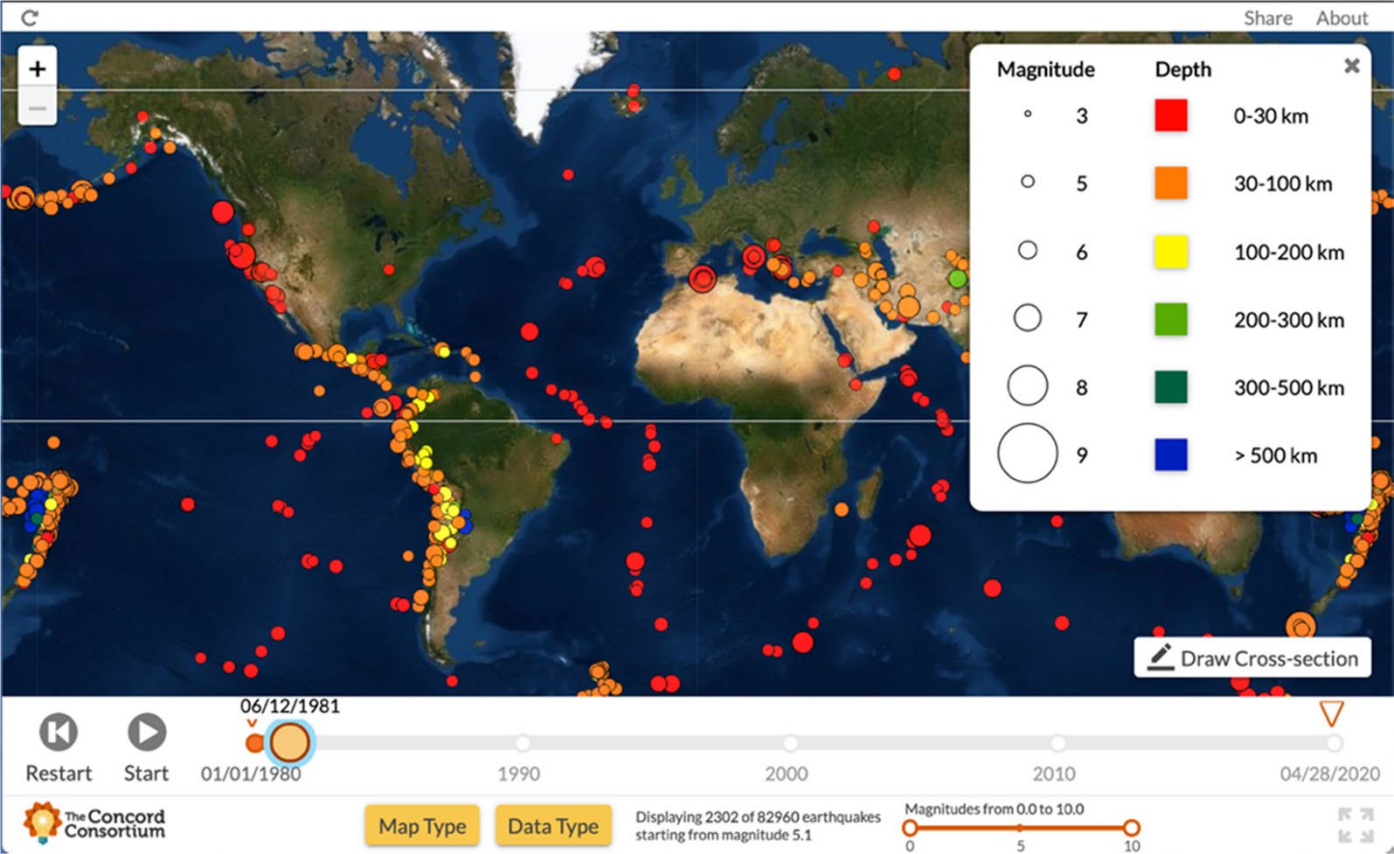
Seismic explorer affordances—earthquake timeline view

**Fig. 2:**
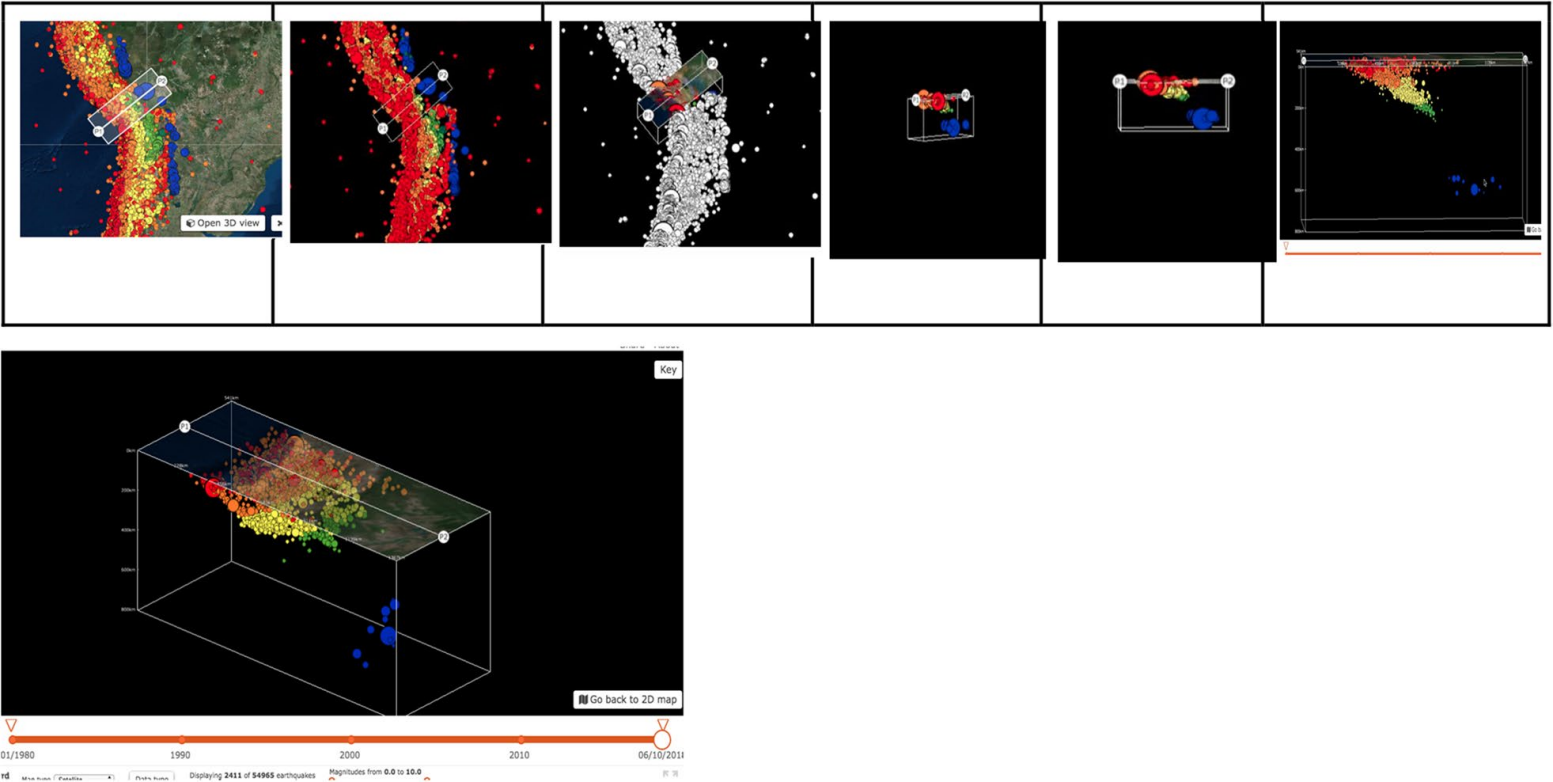
Seismic explorer affordances—dynamic 3D slicing tool

The affordances of Seismic Explorer (Figs. 1 and 2) include a detailed map of the Earth with the ability to overlay three types of data onto the surface—earthquakes (as colored dots), volcanoes (as colored triangles), and plate boundaries (as colored lines). The display can be altered by map types, data types, and earthquake magnitude and time. The visualization has buttons to open or close a key, change map type, change data type, run a time-lapse of earthquake data, and filter for earthquake magnitude. In addition, SE has a dynamic slicing tool that allows users to cut a 3D cross-sectional piece of Earth out of the map to see the pattern of earthquakes below the surface.

The SE visualization has many features to help students visualize the spatial geoscience features of earthquakes, volcanoes, and plates. The analysis of the affordances of the tool demonstrates the interrelationship between this computer visualizations and spatial skills.

## Methods

**Fig. 3:**
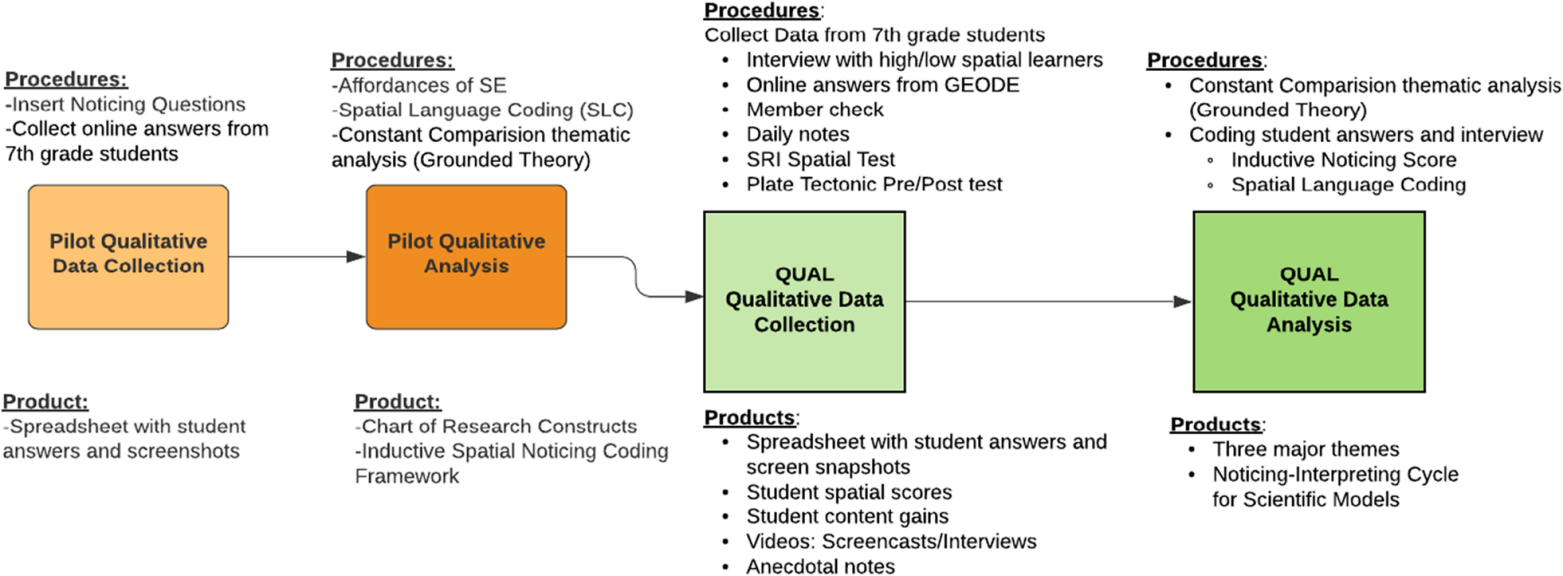
Diagram of methods discussed in this paper: procedures and products. Note. From this research study, 2017

The research project was divided into two parts, a pilot and a main mixed methods study. This paper discusses results from pilot and qualitative portion of main study (Fig. 3).

### Participants

Pilot study—A convenience sample (*n* = 58) of seventh-grade students located in a suburban school district in Pennsylvania near an R1 university participated in the pilot study. The only data collected were artifacts of student work from the online GEODE curriculum. Prior to this unit, students had completed a section of life science curriculum. The data were collected in the spring semester from 12 classes from three experienced teachers.

Main study—A convenience sample (*n* = 119) of seventh-grade students participated in the main study. The students attended one middle school that served a small city in an economically disadvantaged rural area. Plate tectonics content was taught during the fall, after students completed a unit on “measurement and thinking like a scientist.” Prior to the study, the two experienced teachers involved had used a mixture of lectures and hands-on activities followed by assessment in their classes. The teachers taught the plate tectonics module to five classes each.

### Measures

#### SRI test

The Spatial Reasoning Instrument (SRI) was administered to students in the main study prior to the start of the plate tectonic curriculum. SRI was chosen for this research because it is designed for use in research with middle school students (Ramful et al. 2017) and measures three spatial skills—mental rotation, spatial orientation, and spatial visualization—skills needed in STEM curricula typically experienced during ages 11–13. The skills measured by the SRI are related to the skills needed to use Seismic Explorer and to learn plate tectonics (Table 1). SRI incorporates 10 questions from each skill including card rotation tests, cube comparison tests, folding paper tests, and perspective-taking tests and has been tested for reliability and validity. The maximum score is 30 points. Test norms report a score of 10 was in the 10th percentile, a score of 22 was in the 75th percentile, and a score of 28 was in the 95th percentile. Based on these reported parameters and available student population, this study placed students with a score of ten or less as low spatial student and students with score of 20 or more as high spatial.

**Table 1:**
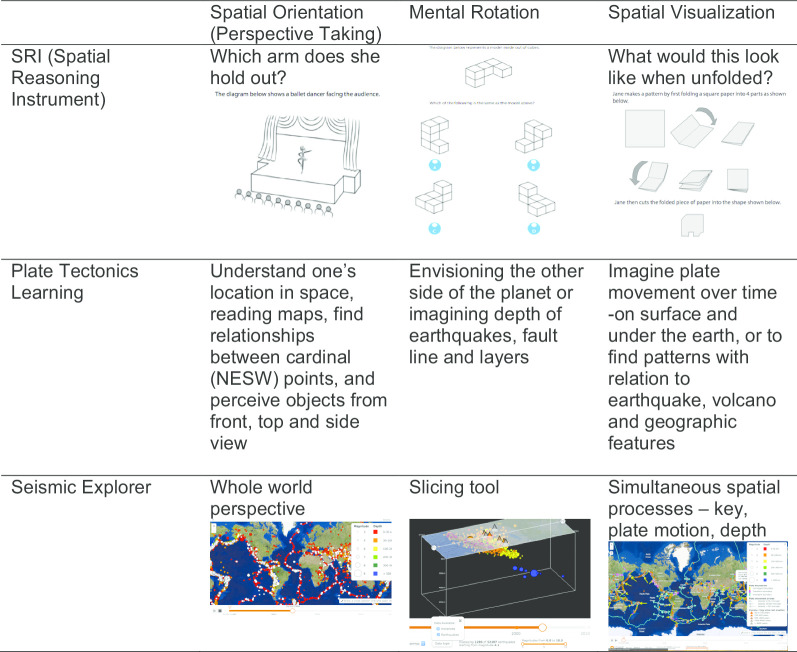
Alignment of Spatial Skills to Plate Tectonics Learning, SE Tools and the SRI Test

From this research study, 2017

#### Plate tectonics content pretest and posttest

A content test developed by researchers by the GEODE project team based on prior research and was administered before and after the curriculum in the main study. The 16-question multiple choice content measure used questions from existing standardized tests collected from outside sources as well as questions developed by the research team. Four of the multiple-choice questions in the online test used questions from the Plate Tectonics Multiple Choice (PTMC) assessment tool. The PTMC discerns students’ level of understanding along various degrees of sophistication to an upper anchor of the plate tectonics learning progression.

Table 1 showcases how three different spatial skills are utilized across three different dependent measures—plate tectonics understandings, the Spatial Reasoning Instrument (SRI), and the affordances of the SE visualization (see Table 1). Table 1 matches the three spatial skills (spatial orientation, mental rotation, and spatial visualization on row 1) with example questions on the SRI (row 2), the use of those skills to support learning plate tectonics (row 3) and how the skills could be used while using the Seismic Explorer visualization.

#### Spatial language coding system

The Spatial Language Coding System (SLCS) was used to code student uses of words—both written and spoken—in both the pilot and main study. SLCS was designed to study overall naturalistic spatial language to determine the frequency of spatial words individuals use across different spatial domains (Cannon et al. 2007). Words were coded in terms of spatial dimensions, shapes, locations and directions, orientations and transformations, continuous amount, deictics, spatial features and properties, and patterns. Spoken and typed spatial words from interviews and digital artifacts were totaled to arrive at a spatial word score for each student. This measure counts the existence of a spatial words so a computer search feature was used to find the number and variety of spatial words in the phases typed by students.

#### Spatial noticing coding framework

In addition to the analysis of spatial words based on the spatial language coding system, a coding framework for spatial noticing was developed as part of the pilot study to classify the spatial references specifically generated while answering four noticing questions in the GEODE curriculum. The first question asks the students to “Describe the pattern of earthquake depth and magnitude in your cross section (of South America). Why do you think the earthquakes form that pattern?”. The next question asks the students to “Describe some features of the topography (the ups and downs of the land surface) of the Indian Ocean”. The third asks “What do you notice about the pattern of earthquakes and the topography of the Indian Ocean? And the last question asks “Describe the pattern of earthquake depth and magnitude in your cross section (of the Indian Ocean). Why do you think the earthquakes form that pattern?

#### Interviews and typed curriculum answers

In addition to the two measures, we also coded student interviews and typed curriculum answers to understand their spatial words and noticing. The student responses to the GEODE curriculum questions were analyzed to distinguish modes of relationships: similarity and contiguity (Maxwell and Miller 2008). Students were scored from 0 to 3: a score of 0 (none) for no or incorrect answers; a score 1 (low) for answers based on colors, a score 2 (medium) for answers based on depth or earth feature, or a score 3 (high) for answers based on combination of depth, earth feature, or plates. The higher the score, the more spatially integrated the students’ written answers were. For example, students who only answered a noticing question using color (score 1) did not provide evidence of using spatial skills. On the other hand, students who discussed depth and related the depth to Earth features (score 3) showed evidence of using spatial skills. Two raters scored the entire set of 64 students’ responses to four noticing questions in the GEODE curriculum. Each question was scored from 0 to 3. The inter-rater reliability was measured using Kappa for each GEODE question: 0.78, *p* < 0.001, for the first question; 0.75, *p* < 0.001 for the second question; 0.58, *p* < 0.001, for the third question; 0.67, *p* < 0.001 for the fourth question. The exact score agreement between the two raters was 88% for the first question, 86% for the second question, 77% for the third question, and 78% for the fourth question. A spatial noticing score for each student was computed by averaging all scores the student received across the four questions.

Student whose answers went beyond the color seemed to indicate that they were beginning to interpret the 3D nature of the phenomena around plate tectonics (Kastens et al. 2006; Newcombe 2016). The Spatial Noticing Coding Framework along with multiple exemplars for each of the coding levels was reviewed and approved by four independent researchers not familiar with the project in an effort to confirm face validity.

## Procedures

This study was conducted as the qualitative study portion of the Pretest–Posttest Control Group design where the GEODE curriculum treatment was randomly assigned at the class level. All students (*n* = 119) took a spatial (SRI) test and a plate tectonics content pretest. Then five classes each received one of two treatments: controlled (*n* = 57; regular classroom plate tectonic instruction) or GEODE (*n* = 62, online curriculum with embedded Seismic Explorer visualization) treatment. Student answer data were collected from the GEODE treatment group only. The control group received an *alternative* activity that paralleled the GEODE curriculum content as agreed upon by teachers. Nine students from both control and GEODE classrooms were interviewed using think-aloud semi-structured interviews (see Table 2). The students interacted with the SE visualization tool (not imbedded in the GEODE curriculum) in the middle of the three-week period they were learning about plate tectonics in school. The primary selection criteria were of students that represented the extreme high and low spatial abilities based on their scores on SRI test. We then tried to balance the interviewed students in terms of high/low spatial scores, gender, treatment/control, and the criterion of being talkative (López and Pintó 2017). After the treatment, both groups took the plate tectonics content posttest that was identical to the pretest. In this study, an unequal sample size was used for the two parts of the analysis. Large classroom student data sets were used for coding student answers to online curricula. Student interviews were limited to a few targeted high and low spatial learners, in order to establish specific comparisons to differentiate between cases (Adams 2008).

**Table 2:** Student interviews

Date NAME	SRI Score	Control or GEODE	Pre	Post	Gain	Sci Grade	Notes
10–18 LENA	7	GEODE	4	5	1	52	Interviewed 2x – pre–pre/post
10–19 DEB	7	Control	3	2	−1	65	
10–25 LYNN	5	GEODE	6	4	−2	84	No screencast, only field notes
10–28 ROY	6	GEODE	1	4	3	58	
11–3 CLINT	8	Control	5	3	−2	71	4 min of video—tech issue
11–1 AYDEN	25	GEODE	9	7	−2	96	
10–23 BOB	25	Control	3	11	8	89	
10–25 LAURI	24	Control	2	5	3	99	
11–3 LYNSEY	27	Control	6	7	1	93	

### Data collection and analysis

#### Measures

Prior to starting the plate tectonic curriculum, student data from the Spatial Reasoning Instrument and the plate tectonic content pre-test were collected online and at the conclusion of the curriculum, all students took the content post-test.

#### Student typed curriculum module responses (GEODE)

Student answers to four noticing questions were selected for analysis. The questions focused on what students noticed about earthquakes in two locations on Earth: the west coast of South America (Peru and Chile), and the basin of the Indian Ocean between Africa and Australia.

#### Student interviews

Due to constraints of testing in the field, students used in the interviews were from both treatment groups. This means that some students would have had experience of using the SE feature before the interview, whilst some would have not. The students were interviewed *during* the three-week period when students were studying earthquakes, volcanoes, and plate tectonics in science classes. Table 2 shows nine interviewed students: four from GEODE classes and five from control classes. Five students scored lower than 10 out of 30 on the SRI test and were categorized as low spatial ability. Four students scored higher than 24 out of 30 score and were categorized as high spatial ability. Student interviews were recorded using screencast software that captured what was happening on the computer screen and the audio of the student’s and interviewer’s voices. Screencast videos were limited in order to establish specific comparisons to differentiate between cases (Adams 2008). Interviews were conducted while GEODE students were using the GEODE online curriculum and control students were doing similar text book curriculum. Students were asked “What do you notice?” while they were interacting with the Seismic Explorer. Screencasts were analyzed at the portion of the interview where students were using a specific spatial skill (Table 3). All interviews were transcribed. The excerpts used in this article were chosen to represent when students are using SE to look at the map from overhead (spatial orientation), using the slicing tool (mental rotation), or interpreting the key to the map (spatial visualization).

**Table 3:** Spatial skills used in seismic explorer

Spatial skill	Definition	Curriculum activity	SE visualizations
Spatial orientation	Ability to find a spatial relationship in reference to a student’s body	Use spatial orientation to interpret the maps from overhead to see height of mountains, depth of ocean, and depth of earthquakes within Earth	
Mental Rotation	Ability to quickly and precisely rotate either 2D or 3D figures	Use mental rotation to interpret the motion of the 3D slice as the slice is pulled out of ground and rotated to show the side of the slice as well as rotated around the axis	
Spatial visualization	Ability to perform a complex spatial procedure with many steps	Use spatial visualization between the key and the map to interpret magnitude and depth of earthquakes, topographical data on land and in ocean, and temporal data as earthquakes are embedded on the map	

## Results

The analysis of students’ typed answers to the noticing questions in the GEODE module and their interviews yielded several differences between high and low spatial students in what they notice, how they interpret the visualizations, and the spatial language they use when describing their interpretations. The three key difference were in ways they talked about depth, identified patterns, and if they proposed explanations or hypothesizes.

### Talking about depths

When talking about the physical dimensions of Earth, high spatial learners used words related to depths and heights while low spatial learners talked about the colors of the features. High spatial learners noticed/interpreted the information from the key that explained the various earthquake depths and verbalized the earthquakes’ depths from the surface. High spatial students seemed to use spatial visualizations and spatial orientations in combination with the information from the map key to develop more sophisticated understandings. For example, these skills would help with noticing that volcanoes only are located over earthquakes of a specific depth. Low spatial learners eventually discussed depths, but only after first verbalizing they were seeing colors. Low spatial learners seemed unable to analyze the images on the SE to make proper interpretations of the earthquake data, and thus did not get to the level of making inferences.

**Fig. 4:**
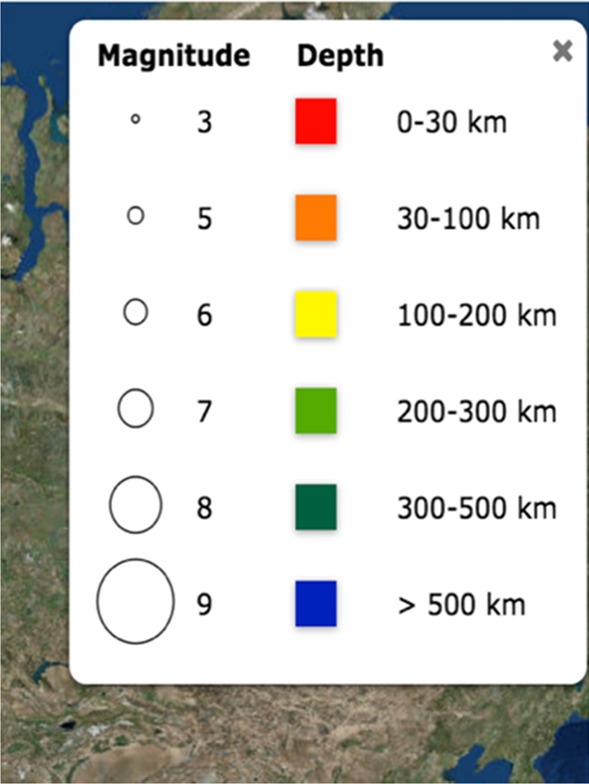
Seismic explorer earthquake key

In SE, the dots located on the seismic activity map to represent the earthquakes had two meanings. According to the key, the dot had a color that was meant to indicate the depth at which the earthquake occurred. In addition, the size of the dot indicated the magnitude of the earthquake. The key showed how the color and the size of the dot should be interpreted (Fig. 4). This pairing of two types of information resulted in misinterpretations by the students when they conflated the unique meanings of the dots’ size and color. To connect the SE visualization to the Earth-related phenomena, students would likely need to use spatial visualization skills to connect information from the key to data in the map.

Throughout the interviews, there were clear differences in the ability of high spatial learners to notice relations between colors and Earth features compared to low spatial learners. For example, here is an episode where a high spatial student, Bob (control), looked at the first screen with earthquake data embedded on the world map, with the key on the screen (See Table 3c). The earthquakes are represented as colored dots; the key explains that different colors represent different depths.

Researcher: So, what else with regard to the key do you see here? [looks at earthquake data on the map and key that relates to the data].

BOB: It looks like that there is less depth when it is above oceanic crust. Umm—yeah cause most depth was along inland.

Here Bob appeared to use visual orientation to notice that the earthquakes above oceanic crust were shallow compared to those along inland, as opposed to stating they were red. He correctly interpreted the red color from the key to relate the depth of the earthquake to the geological location of ocean as compared to land. He also discussed the geologic feature of the ocean crust, meaning he interpreted an array of the colored dots in relation to the landforms represented on the map. So, Bob’s initial words were after he mentally interpreted the key’s colors to be depths and noticed the features represented on the maps. During other interviews, all of the high spatial learners expressed understanding of the earthquakes in terms of depths instead of colors, regardless if they were using SE in class or not.

On the other hand, a low spatial learner Roy (GEODE) needed more scaffolding prompts from the researcher to process the key’s meaning. In the episode Roy looked at the earthquake visualization just before the earthquake data is embedded on the world map (see Table 3a).

R: Go ahead and press the play button and when that is playing, what does that, what do you see? [the simulation starts embedding colored dots that represent earthquakes on map over time].

ROY: Umm, it shows all the earthquake activities.

R: How does it show it?

ROY: As like different colors for different sized deep, it has different colors for like other deep depths of hurricanes.

Roy initially explained that he saw earthquake activities, then talked about color, and finally mentioned hurricane (he likely misspoke) depth. In contrast to high spatial learners, he took three statements to finally talk about the spatial property of earthquake depth. Also, Roy did not relate the earthquakes to the earth’s features during this interchange. In fact, all low spatial interviewees pointed out the color first and then discussed the meaning of the color. Only two of the four low spatial students interviewed correctly interpreted the color as the earthquake depth. Interview results like these suggest high spatial learners can notice the relationship between the key, the landforms on the map, and the interpretation of the computer visualization in terms of the depth of the earthquakes, while low spatial learners need more interactions or scaffolding from the researcher to express these relationships or even identify the depth information.

### Identifying patterns

High spatial learners were also more likely to notice patterns and often went into more sophisticated interpretations of the visualization. SE allowed students to view earthquake data in 3-D on a 2D computer screen. High spatial students seldom had trouble seeing the dimensions of the data while low spatial students struggled, especially with the ocean base maps. On the SE maps, different color schemes were used to convey information about heights of lands and depths of ocean. First, the shadowing on the land masses was used to display different land heights around mountains. Ocean depths, which were a kind of inverse of the land heights, were shown using variations of blue. Finally, the color and size of the earthquake dots represented depth and magnitude of earthquakes both within and under the continental and oceanic crusts. Students needed to use spatial orientation skills to comprehend the scope and variety of depths presented on the computer screen in three different color schemes and the relationships among landforms (continental and oceanic crusts, mountains, and ocean) and earthquake events. Being able to use spatial skills to integrate all this information was needed to notice and interpret patterns on the screen.

SE visualizations appeared to support both types of spatial students to notice patterns in the earthquake and volcano data, although high spatial students made more sophisticated connections between the map and the earthquake patterns, and used more technical scientific words like trench and continent. The following episodes highlight how students see patterns in different ways.

**Fig. 5:**
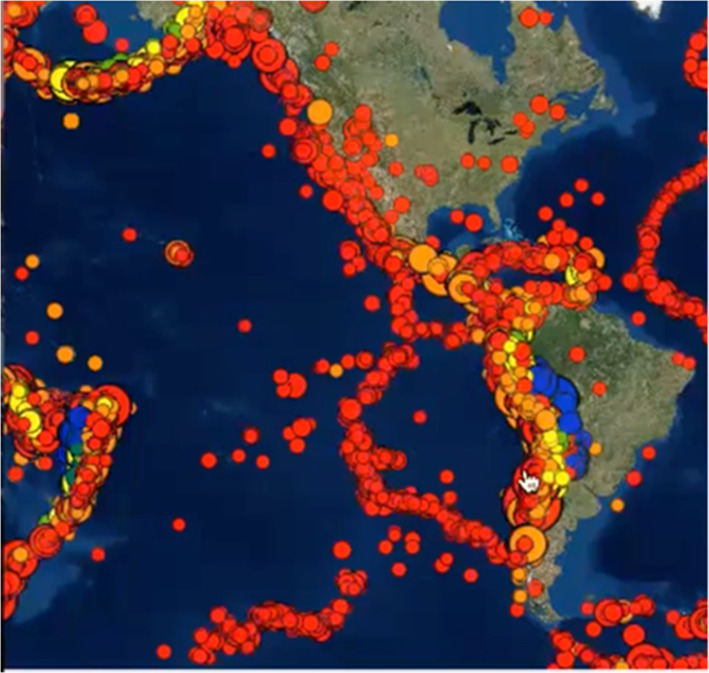
Excerpt of Screencast with Bob From this study data files, 2017

Bob (control, high spatial), who was using SE to look down on the earth from above the surface, was examining the earthquake data embedded on the map of the world (Fig. 5). He made three observations about the earthquake patterns.

R: So, as that is playing, what are you seeing?

BOB: That they are consistent in the same spots. It looks like there is consistent looping of where the earthquakes are. Especially right along the trench here. [puts pointer on the trench along the west coast of South America].

Bob noticed the patterns of the earthquakes being in the same location on the map, that earthquakes recur on the same location, and that the earthquakes line up along the trench. This observation seems to indicate spatial orientation observation, as he first had to understand that the ocean-base map depicted a trench at the same location as the earthquakes. Three out of 4 high spatial students interviewed were able to notice the patterns between earthquake locations and specific landforms from the visualization. Such pattern recognition between earthquake locations and landforms is critical in understanding the plate tectonics theory. Earthquakes occur as two or more plates move toward or move away from one another. Particular landforms such as ocean trenches and mid-ocean ridges are formed at plate boundaries depending upon how two plates move.

In contrast, low spatial students did not report observing as much detail in the patterns as high spatial students. For example, Deb (control) was looking at the SE map just prior to embedding the data on the map (Table 3a). Once the data were on, Deb did not directly report seeing a pattern but did explain what the dots represented as a function of the key.

R: So. what happens when I press this [play] button… I will start it and you can tell me.

DEB: Umm, earthquakes appear in certain parts of the map and when they are popping up is when it tells us that what their, like, depth is and their strengths and magnitude and where they occurred.

In this episode, Deb never mentions a pattern in the earthquakes in relation to landforms, but more generally indicates “certain parts of the map.” This term could mean she saw a pattern, or simply that she saw the dots were scattered across the map. She never talked about locations on the map, or related the earthquake data to Earth features, and she gave no evidence of using spatial orientation. In similar episodes, it was not clear whether low spatial students did not notice patterns that were meaningful to understand plate motions. Lena (low) was not sure she saw earthquake patterns across the surface of the Earth, while Ayden (high) noticed a pattern in the earthquakes in a 3D slice and suggested it indicated subduction with the earthquakes occurring on a descending slab (Table 3c). High spatial learners noticed the interrelation between the visualization features (e.g., color and size of dots), patterns of phenomena (e.g., earthquakes), and landforms. Low spatial students seemed less confident in their answers, sometimes seeing only patterns in the visualization, sometimes being able to link the images on the screen to the earthquake patterns, but rarely connecting either of these patterns to landforms without guidance and prompting. The ability to see patterns in data is an important skill in science, particularly so in systems-level geoscience such as plate tectonics.

### Making hypotheses or explanations about data

**Fig. 6:**
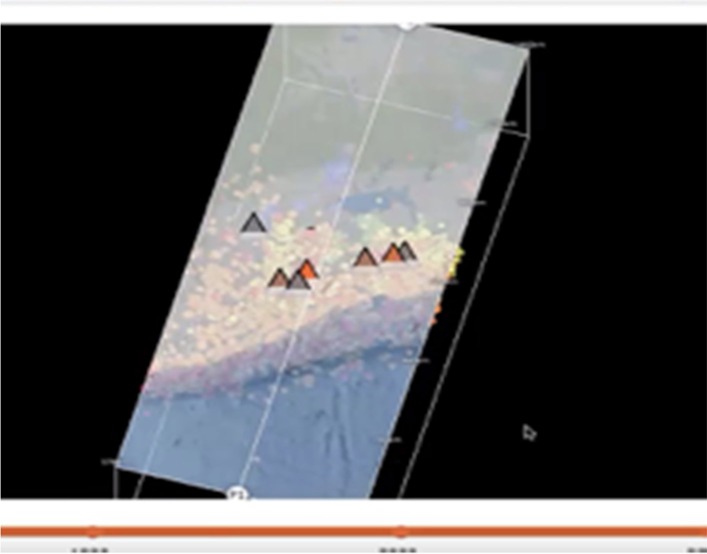
Excerpt of Screencast with Lynsey from this study data files, 2017

High spatial students made inferences in the form of hypotheses or explanations possibly based on their spatial understanding; low spatial students merely described what they saw. The following episode from the interviews reveal how the high spatial learners went beyond the visualization and tried to interpret the data. Lynsey (control), a high spatial learner, correctly noticed a pattern between the depths of earthquake and the location of volcanoes (see Fig. 6).

R: Now you can actually move this around now. [demonstrates the motion of the 3D block] So now tell me what you are seeing. It only goes about 180 or something like that.

LYNSEY: [moves block around] There aren’t that many major earthquakes, well maybe deep ones, but there are very few down here. And a lot of, up here, [moves block so she can see the top] there seems to be a lot of little minor things, it looks like there is more volcanoes on the slightly deeper earthquakes. Like not the red ones, but the orange and yellow ones.

In this episode, Lynsey showed a comfort with the idea of relating color to depth by using the terms interchangeably. She also understood the magnitude was represented by the diameter of the dot, correctly discussing the general lack of major earthquakes except the “deep ones.” She did not use the word blue to discuss the deep earthquake, but only referred to earthquakes being either deep or slightly deeper than the red one. Perhaps her spatial visualization and mental rotation skills allowed her to recognize that the earthquakes had different depths and magnitudes based on the colors and diameter of the dots. Furthermore, she correctly discerned that the earthquake depths were associated with the presence of volcanoes which allowed her to make inferences about the visualization.

Among the nine students interviewed, three of the four high spatial students took the data and made an inference or developed a hypothesis about the meaning of the data. There were no instances of low spatial learners (even among students who were using SE in class) interpreting or inferring ideas about the meaning of the earthquake data or its relationships to other data or aspects of the visualization. High spatial students have enough understanding of the visual representation to use some of their observations about the location of volcanoes and other landforms to point out related phenomena that occur around plate subduction zones. Part of developing a more sophisticated understanding of plate tectonics is being able to see patterns and then use these patterns in the data as evidence to support the claims in the context of complex visualizations. Higher spatial students were able to progress further in their line of reasoning, whether they were familiar with SE from class or not, because they were able to notice important details in the data and connect patterns across multiple aspects of the visualization.

The interviews exposed differences between high and low spatial students by the way they talked about depth, recognized patterns and made hypothesis or explanations. In addition, there were also difference in the quality and quantity of spatial words used in the interviews and in typed answers.

### Using spatial words

Analysis of interview transcripts and students’ typed answers to the four noticing questions in the online curriculum module revealed that students with low spatial skills did not talk or type with as many spatial words as those with higher spatial skills.

#### Eight student interviews

**Fig. 7:**
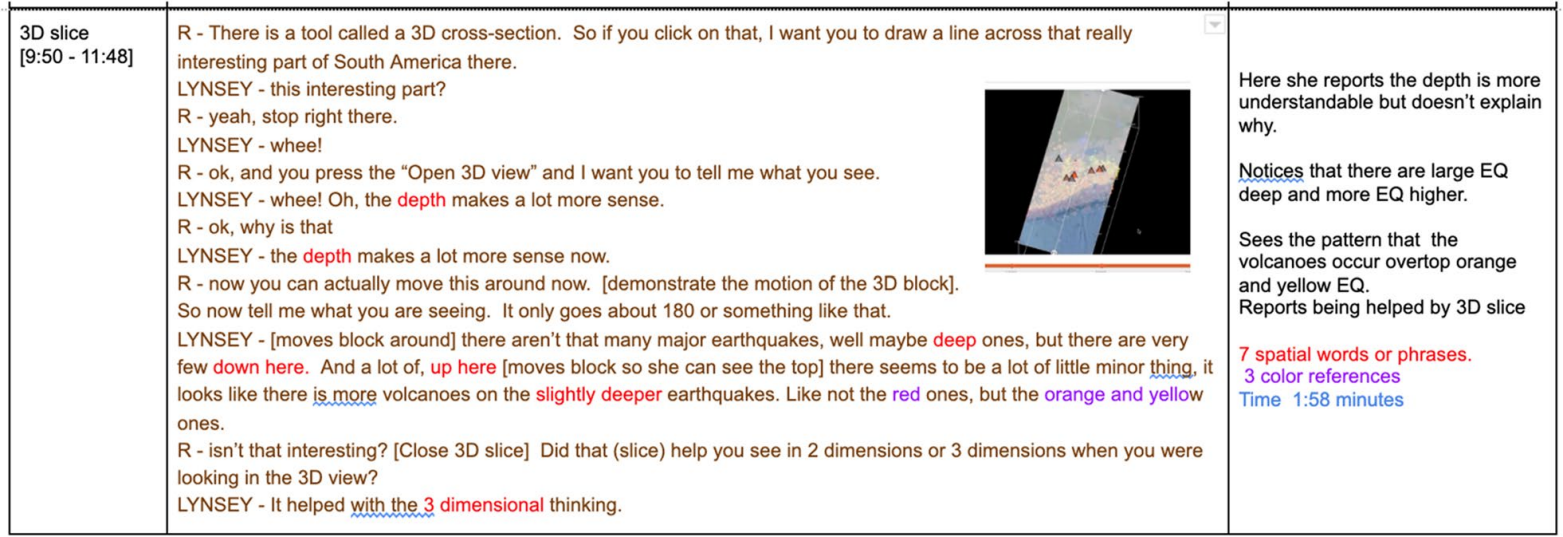
Sample spatial coding using inferential spatial language coding or color

High spatial students talked more often using spatial terms than did low spatial students. Table 4 shows descriptive statistics on the length of interview, the number of spatial words, the number of color words, and the ratio between spatial and color words. The interview segments where these words were counted were related to the key with embedding earthquakes, ocean base map, and 3D slicing tool. Words that described colors (red, orange, blue, etc.) and the word color were coded as colors (Fig. 7). Words that fell into the spatial language coding (Cannon et al. 2007) were coded as spatial. Each repetition of a spatial word counted as one contribution to the total spatial word count. Words from each section were summed up and ratios of spatial words versus color references used by student when discussing the SE visualization were calculated (Table 4).

**Table 4:** Ratio of descriptive words used in interviews—spatial to color

Student name and (spatial score)	Length of Interview (mins)	Number of spatial words	Number of color words	Ratio (spatial: color)
*(a) High spatial students*				
LAURI	14:03	24	5	4.8: 1
LYNSEY	5:49	13	3	4.3: 1
BOB	13:16	31	1	3.1: 1
AYDEN	9:31	17	5	3.4: 1
Average	10:39	21	3.5	3.9: 1
*(b) Low spatial students*				
ROY	4:25	11	4	2.8: 1
CLINT	2:12	4	4	1.1: 1
DEB	4:42	17	12	1:4: 1
LENA	13:27	25	9	2.8: 1
Average	6:19	14.25	7.25	2.0: 1

From this small data set, the ratio of the average number of spatial descriptions to color descriptions for high spatial skill students is 3.9 spatial words for every color word. Low spatial students used spatial descriptions on average at a rate of 2.0 spatial words for every color word. High spatial students used spatial words almost twice as often as low spatial students (3.9:1 vs. 2.0:1), with the extreme high spatial (Lauri) more than four times greater than the extreme low spatial (Clint).

#### Students’ typed spatial answers (GEODE class)

**Fig. 8:**
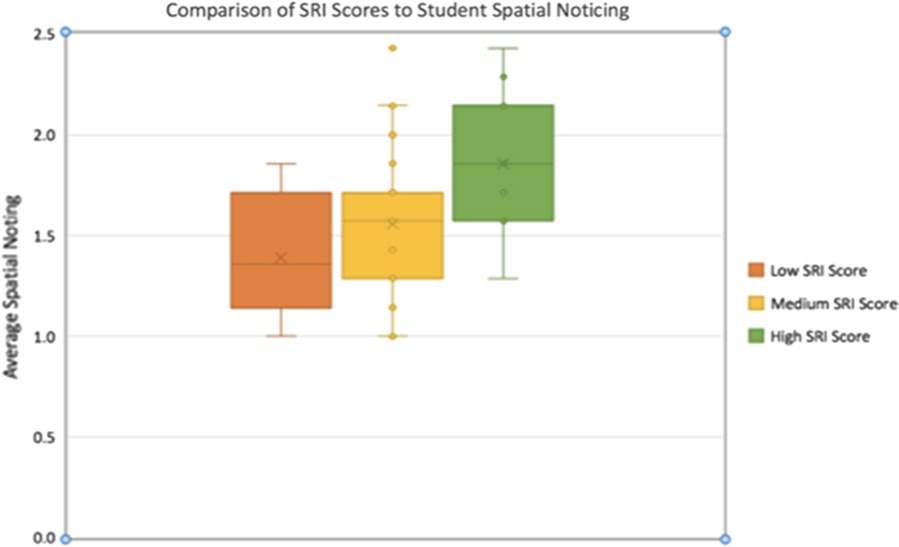
Average noticing spatial answer score compared to SRI score range

Student typed responses were coded using the Spatial Noticing Framework developed in the pilot. Based on SRI test scores, students who were in the GEODE treatment (*n* = 64) were categorized into low (*n* = 17, scored between 0 and 10), medium (n = 33, scored between 11 and 19), and high (*n* = 14, scored between 20 and 30) spatial ability groups. Students’ answers to four questions in the curriculum module were scored from 0 to 3 using the framework from the pilot. Spatial Noticing scores from the four curriculum questions were added to create a total spatial noticing score for each student. Figure 8 shows the box plot of the averaged noticing score across three spatial ability groups. The average spatial noticing score was 1.38 (SD = 0.32) for the low spatial ability group; 1.56 (SD = 0.35) for the medium spatial ability group, and 1.87 (SD = 0.32) for the high spatial ability group. ANOVA indicated that the mean differences among the three groups were statistically significant, F(2,59) = 7.67, *p* < 001. Tukey’s post hoc analyses showed that the mean of the high ability group was significantly higher than that of the low ability group, ES (Cohen’s *d*) = 1.53 SD, *p* < 0.001, and that of the medium ability group, ES (Cohen’s *d*) = 0 0.91 SD, *p* < 0.05.

These findings indicate that high spatial students expressed their understanding of earthquake/volcano phenomena using more spatially oriented descriptions than low and medium spatial students, indicating the importance of spatial abilities in learning about tectonic phenomena based on spatially rich visualizations.

These results from the analyses of the interviews of selected students using Spatial Noticing coding and the typed answers of students who engaged in the curriculum module using Spatial Language Coding showed similar trends. Students with high SRI scores used a greater number of spatial words than students with low SRI scores. Students with high spatial skills exhibited more sophisticated plate tectonics understandings by linking earthquake location, depth, and magnitude information inferred from the location, color, and size of circles to geospatial features such as mountains and ocean trenches. Low spatial students however used more descriptive, non-spatial language to express their understanding at the surface level representation.

## Discussion

In this study, there were differences in the way high and low spatial students noticed and interpreted seismic activity data in the visualization. These differences can be associated with more or less understanding of science content. In particular, the differences that arose in the interviews include the way students talked about depth of earthquakes and how they recognized patterns. Interviews with students on the extreme ends of spatial skills scores also revealed that higher scoring students (> 20) on the Spatial Reasoning Instrument (SRI) noticed and interpreted more details in the data while using the computer visualization than lower scoring students (< 10). High spatial students saw more patterns and made more inferences and hypothesis about what they saw in the computer visualization compared to low spatial students. Differences were also found in the written and spoken words of students, where high spatial students used a larger ratio amount of spatial words in their explanations.

**Fig. 9:**
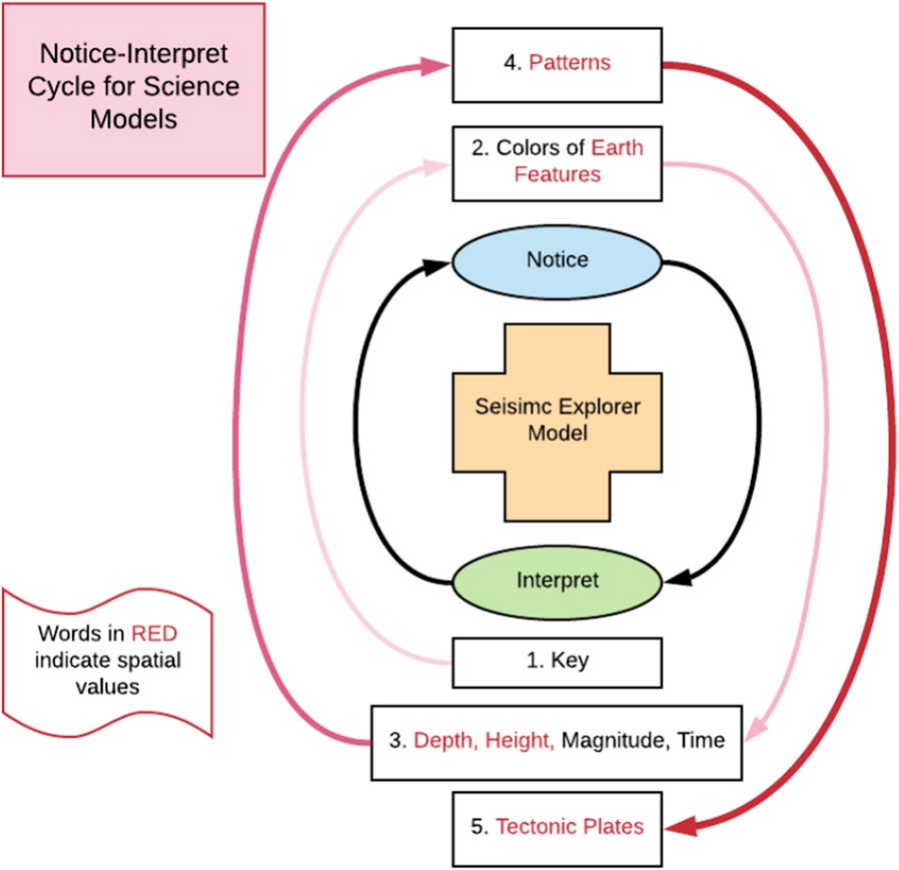
Notice–interpret cycle for seismic explorer visualization

The results show a positive relationship between a student’s SRI scores (Ramful et al. 2016) and what they notice-interpret in the computer simulations and ultimately their ability to gain sophisticated science understanding of plate tectonics. The overall pattern suggests that that students go through an iterative cycle of noticing (Lindgren and Schwartz 2009) and interpreting when using a scientific visualization, continuously cycling between the two processes while viewing and making sense of the visualization. In fact, rather than a cycle, this iterative notice-interpret pattern can be viewed as a spiral, in which students’ interpretations become more sophisticated as they progress through each successive cycle (See Fig. 9).

For example, when using the Seismic Explorer visualization, students must *interpret* the key in terms of color and what it represents; then *notice* the corresponding colors of Earth features on the map; then *interpret* the depth, magnitude, height, and time on the map in relation to the key; then *notice* the patterns on the map; and then *interpret* the patterns in relation to the plates (see Fig. 9). This is a complex iteration of noticing specific salient aspects of the visualization, interpreting them, and then using those interpretations to notice new salient aspects of the representation. And in this case, to what extent the entire iterative cycles could be completed depends on students’ spatial skills.

This iterative notice–interpret can cycle can explain why low spatial students tended to report only descriptions of surface features of the seismic activity visualization, while high spatial students developed more sophisticated interpretations of the seismic data spatially, which allowed them to make inferences. For example, low spatial students should be able to see the colors on the key (step 1 in Fig. 7) and on the map (step 2); however, they may not be able to use spatial visualization to interpret the color as depth of earthquakes (step 3). In addition, they would not be able to notice the earthquakes are aligned along trenches, mountains, and ridges (step 2), because they may not have spatial orientation skills to interpret the heights of the mountains and depths of the trenches (step 3). The last two steps of the example in Fig. 7 are both spatial, where the student would likely use mental rotation skills to notice the earthquake pattern underground (step 4) in order to interpret the location of subduction zones (step 5) of the oceanic plate. Without proper scaffolding of instruction, low spatial students would have a difficult time rising above the first iteration of the cycle, as indicated by low spatial students talking about the earthquake data in terms of colors instead of depth. This finding also explains why high spatial students were able to notice details of the representations, as when Lynsey pointed out that the volcanoes were located above the deeper earthquakes along the plate boundary.

Findings from this study add to the research on spatial skills and STEM success that reports spatial skills can be used to predict STEM talent (Lubinski 2010), spatial skills can be a gateway or barrier into STEM fields (Uttal and Cohen 2012) and spatial skills play a critical role in becoming a STEM expert (Wai et al. 2009). This research shows that students with high spatial skills were able to develop more plate tectonics knowledge through continuously noticing and interpreting increasingly sophisticated features of the visualization at higher and higher inferential levels, and ultimately to develop richer sophisticated understanding than low spatial students. If this trend continues with other visualization-rich domains, high spatial students will end up having greater success in STEM courses and careers because they can better navigate in the spatial world of science, math, and engineering.

This study also supports the idea of the “ability-as-enhancer” hypothesis (Huk 2006; Mayer and Sims 1994) for computer visualizations. This hypothesis expects that students with higher spatial skills will be able to learn more from the visualization because the computer lowers the cognitive load for the high spatial student, allowing more mental model building. In this study, high spatial students appeared to use spatial orientation to navigate complex Earth and ocean maps; use mental rotation to comprehend earthquake and volcano patterns; and use spatial visualization to interpret the key to understand the earthquake and volcano data. Low spatial learners, even though they said the 3D slice of the visualization provided assistance, were more likely to report that they were confused or were not able to verbalize what they were noticing.

This study also adds a new notice–interpret cycle. This cycle identifies how students must spiral between noticing and interpreting the visualization to build their spatial understanding. As spatial understanding grows, so does content knowledge when that involves spatially-oriented concepts. Researchers can use the notice-interpret cycle to monitor student progress toward spatial understanding of the complex science content such as plate tectonics. The notice–interpret cycle can help designers of curriculum, visualization, and professional development programs consider how to support students to use scientific visualizations in the classroom so that they can grow in spatial and science content knowledge.

## Conclusion/limitations

This research centered on extremes of spatial skills, high and low, for the interviews. Transcripts were analyzed around portions of the student interviews that specifically dealt with the spatial skills of spatial orientation, mental rotation, and spatial visualization. A potential confounding variable is that the interviews were held with both control and GEODE groups and there was an unbalanced split within the high spatial group (3 control group, 1 GEODE). Thus, the students came to the interviews with different classroom experiences. Despite the different exposures, the results remained intact. More research could be done to better understand how students with medium spatial skills talk and type with regards to spatial words and what they notice-interpret. Another avenue to pursue would be evaluation of the other spatial skills and compare the results to student demographics, content gains, and noticing. Perhaps one type of spatial skill would stand out as the key skill to train in order to have success with interpretation of the visualization. Also, more think-aloud interviews should be conducted with all types of learners—low, medium, and high spatial—as well as different demographics including English language learners, special education, and socioeconomically disadvantaged students to see if the notice-interpret cycle is relevant for all learners. Another possible interesting future direction of research might be to investigate the spatial types/tokens ratio, to provide an idea of the relationship between spatial skill and the richness and range of spatial language used, to complement the number of spatial words/ratio of spatial to color words. Finally, some of the reported differences could be contributed to other cognitive skills that were not tested (Kozhevnikov et al. 2002) and should be included in future studies.

More research is needed to find to ways to scaffold the curriculum in order to teach the spatial skills needed to comprehend the digital visualizations on the computer screen. Additional research is also needed at the intersection of the spatial words used by the student and the spatial skills of the student, and how the spatial language used in curriculum or by the teacher is related to student learning. Finally, more research is needed in defining best practices for presenting spatial phenomenon on a computer to students – especially students who have low spatial skills.

Besides research, there are considerations for designers, curriculum writers and teachers. When designing a visualization or simulation, designers need to consider age (Lubinski 2010; Piaget and Inhelder 1967), spatial training, cognitive overload and how the scientific model is portrayed (Mayer and Moreno 2003). Specifically, for students with low spatial skills, initial computer models that are presented need to be simplified with fewer features to avoid cognitive outload. Once students display a notice-interpret cycle with the simple model, then more complex models can be presented. Curriculum writers need to consider students’ spatial skills, lack of expertise, and knowledge of the visualization in curriculum development (Davenport and Glaser 2002). Finally, teachers need to consider how students’ differing spatial skills may affect their classroom performance and recognize that visualizations do not necessarily solve plate tectonics misunderstanding.

This research study was an attempt to get a better understanding of how middle school students’ spatial skills can help or hinder their learning of plate tectonics when using computer-based visualizations. The research found that students with high spatial skills incorporate more spatial words in their talks, go through multiple notice and interpret cycles with complex visualizations, and ultimately gain more robust content understanding than low spatial students. Findings of this study suggest that students with higher spatial skills developed more sophisticated understanding because they were able to notice more spatially oriented features of the phenomena presented in the visualization and interpret them in the context of targeted scientific knowledge. More research is needed to fully develop the prosed notice–interpret cycle for scientific visualizations, use spatial words to better understand student learning and determine ways to scaffold low spatial students when using computer-based visualizations.

## Data Availability

The datasets used and/or analyzed during the current study are available from the corresponding author on reasonable request.

## Abbreviations

NGSS: Next-generation science standards
SE: Seismic explorer
SRI: Spatial reasoning instrument
